# A Missed Case of Synovial Osteochondromatosis

**DOI:** 10.7759/cureus.48498

**Published:** 2023-11-08

**Authors:** Omar Alkhabbaz, Yasser Bibi, Brian Webb, Madison Milhoan

**Affiliations:** 1 Texas College of Osteopathic Medicine, University of North Texas Health Science Center, Fort Worth, USA; 2 Orthopaedic Surgery, John Peter Smith Hospital, Fort Worth, USA

**Keywords:** synovectomy, chondromatosis, chondrosarcoma, synovial chondromatosis, osteochondromatosis

## Abstract

Synovial osteochondromatosis or synovial chondromatosis is a benign joint pathology characterized by the development of multiple cartilaginous nodules or loose bodies in the synovial membrane that typically arise in the larger joints of the body. It usually presents as joint pain and, as seen in the present case, can occasionally be missed. Diagnosis involves a combination of clinical evaluation, imaging studies, and histopathological evaluation (which is confirmatory). Treatment depends on the severity of the disease, symptoms, and the patient’s social situation, and may include monitoring for asymptomatic cases, non-surgical management (pain medications and physical therapy), or surgical intervention. Surgical intervention may include arthroscopic removal of loose bodies, synovectomy, or reconstruction/replacement in severe situations. Prognosis has a direct relationship to how early the disease is diagnosed. Early intervention with appropriate management can help alleviate symptoms; however, if left unmanaged, it can lead to joint damage and osteoarthritis or very rarely, malignant transformation into chondrosarcoma. This report describes the case of a 62-year-old female with complaints of bilateral knee pain who was originally diagnosed with osteoarthritis based on clinical exam and X-rays. Two magnetic resonance imaging (MRI) scans were done three years apart, with synovial osteochondromatosis being on the differential after the second scan. A left knee major synovectomy was conducted after the second MRI reading, where rubbery masses of tissue along with loose fragments were removed. Fluid from the tissue masses was sent to culture and pathology for interpretation. Two weeks post the surgery, the patient’s pain improved tremendously, with adequate ambulation independently. Histopathology came back positive for synovial osteochondromatosis. This case report highlights the importance of keeping this joint pathology on the differential when treating patients with joint pain and interpreting imaging.

## Introduction

Synovial osteochondromatosis is a benign, uncommon, and interesting disorder that presents challenges in diagnosis and treatment. It is a complication of the synovial membrane of joints, tendon sheath, and bursae that can cause dysfunction most commonly in the knee, hip, or shoulder that has the potential of transforming into chondrosarcoma [1]. While the exact etiology of a primary synovial osteochondromatosis is still in question, hypotheses point toward the metaplasia of the synovial membrane and the detachment of cartilaginous fragments as potential causation [2]. The cause of secondary synovial osteochondromatosis is associated with an underlying joint pathology or condition that triggers development such as osteoarthritis, rheumatoid arthritis, or traumatic joint injuries [2]. Patients with this condition usually present with a history of pain in the joint affected, swelling, crepitus, and limited range of motion (ROM) [2]. The pain is usually aching in nature and is exacerbated by weight-bearing activities such as prolonged periods of walking or standing [2]. 

Diagnostic imaging aims to confirm the presence of the condition, evaluate the severity, and rule out underlying pathologies. Plain radiographs (X-rays) are often the initial imaging modality and can be used to identify joint effusion, loose, bodies, or potential calcifications in joint space. However, plain radiographs may only sometimes be accurate because, in 20% of cases, calcification and mineralization are delayed and can often take years to develop [3]. Computed tomography (CT) may be utilized to provide additional information such as the presence of loose bodies, joint degeneration, or bone erosions [4]. Magnetic resonance imaging (MRI) can detect synovial hypertrophy, joint effusion, cartilaginous nodules, and loose bodies [5]. Definitive diagnosis (gold standard) requires a histopathological examination, where tissue samples are obtained from the synovial membrane or loose bodies of the affected joint [2]. This will typically show synovial hyperplasia, chondrocyte clusters, loose bodies, fibrosis, and inflammatory changes on histology. 

During the early stages of the disease, conservative management can be attempted with the use of non-steroidal anti-inflammatory drugs (NSAIDs) to reduce any swelling around the affected joint(s) [6]. However, once loose bodies are detected, surgical intervention is generally necessary to prevent the restriction of joint movement and persistent swelling [6]. The condition is most commonly managed with arthroscopic loose body removal with synovectomy to remove any of the affected synovium [7]. Depending on the extent of the condition, open radical synovectomy may be recommended for management to prevent recurrence in extensive involvement [8]. The benefit of performing a complete synovectomy has been debated as it may be associated with increased morbidity; however, the procedure decreases the risk of recurrence [9]. Nonetheless, the removal of loose bodies with synovectomy remains the treatment of choice as the pathogenesis of synovial osteochondromatosis is believed to be due to the metaplastic activity of the synovium [9].

## Case presentation

A 62-year-old female with a past medical history of hypertension and hypothyroidism presented to the clinic with a chief complaint of bilateral knee pain and effusions, which was diagnosed as osteoarthritis based on clinical exam and past X-rays. The patient had been experiencing moderate relief from bilateral steroid injections that were administered approximately every four to six months. As for diagnostic imaging, she had two MRI scans of her left knee; the first in 2020 and the second in 2023.

**Figure 1:**
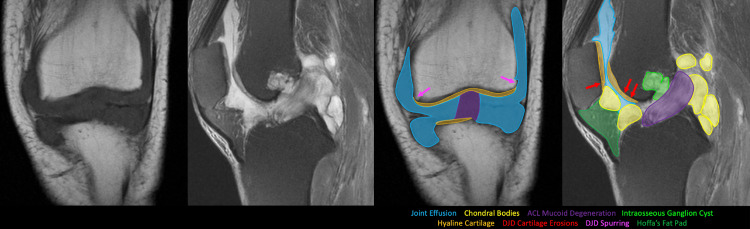
Annotated Pre-Operational MRI images

Although the MRI scans did not produce a differential of a synovial osteochondromatosis, the one in 2023 was interpreted by the radiologist as showing mucoid degeneration of the anterior cruciate ligament (ACL) with multiple periligamentous and intraosseous ganglia, a large joint effusion, and chondral abnormalities. As for the articular cartilage, there was a small cartilage fissure overlying the medial patellar facet. There was also a small high-grade cartilage defect overlying the medial femoral trochlea with a possible cartilage flap. The differential diagnosis formulated in the clinic after the MRI scan (Figure 1) was pigmented villonodular synovitis (PVNS), rheumatoid arthritis (RA), or synovial chondromatosis. 

**Figure 2:**
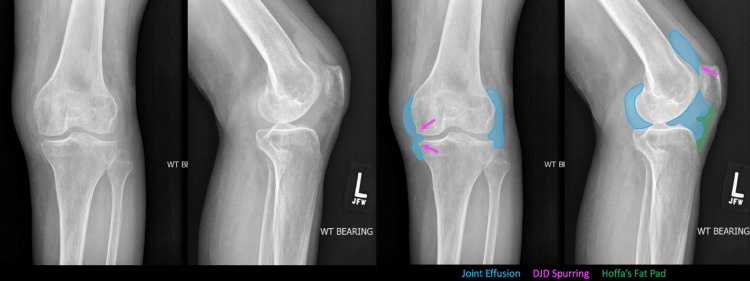
Annotated Pre-Operational X-ray

These findings warranted a diagnostic left knee arthroscopy as shown in Figure 2, with a synovial biopsy for evaluation.

**Figure 3:**
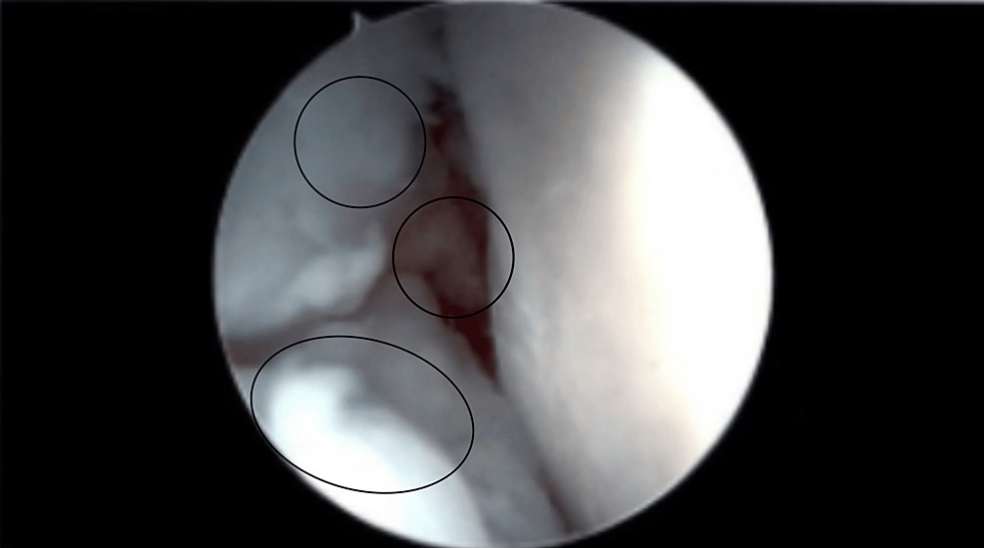
Intra-Operational Arthroscopy Image Showing Foreign Bodies in Medial Gutter

**Figure 4:**
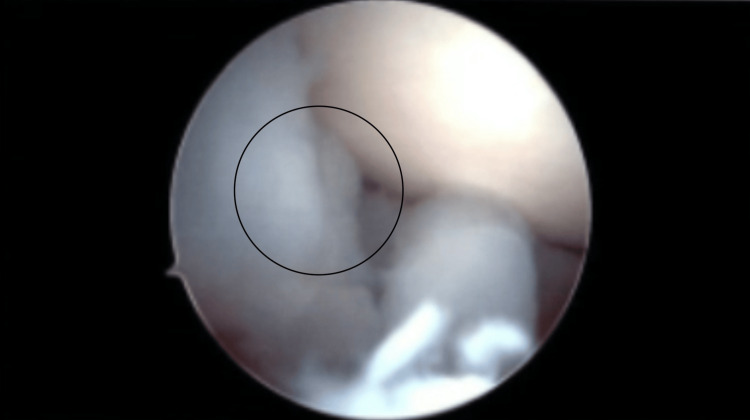
Intra-Operational Arthroscopy Image Showing Foreign Bodies in Intra-Articular View

**Figure 5:**
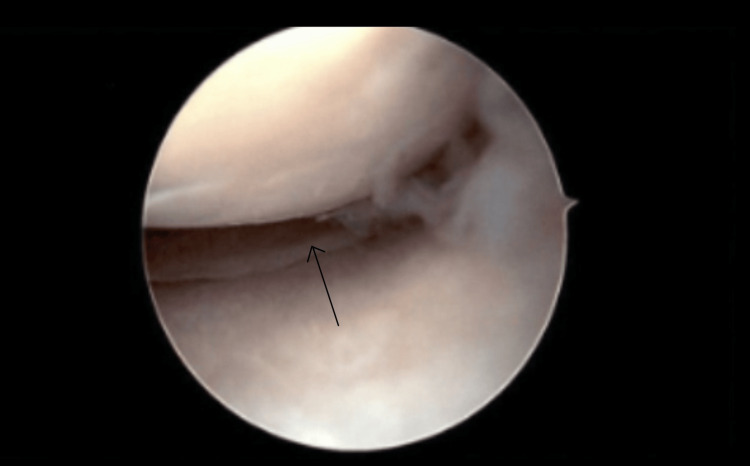
Intra-Operational Arthroscopy Image Showing Intact Menisci

A left knee major synovectomy took place. Anteromedial, anterolateral, and superolateral portals were made with a knife blade. The anteromedial portal was placed under direct vision. The patellofemoral joint had grade three arthritic changes of the patella. There were also grade three arthritic changes of the trochlea. As for the gutters, they were full of white rubbery masses of tissue (both sides of gutters, notch, and medial and lateral compartments). Figure 3 shows the foreign bodies present in the medial gutter. Figure 4 shows an intra-articular view of the foreign bodies. These were pulled out one by one and debrided with a shaver and wand. Fluid was sent for culture, cell count, and pathology for all these loose fragments. The medial compartment did not contain a medial meniscus tear. There were grade one arthritic changes of the medial femoral condyle and medial tibial plateau. A synovectomy was also performed here as loose fragments were present. The notch region had the ACL intact with the probe; however, synovectomy was conducted here as the ACL looked degenerative as seen in the MRI. In the lateral compartment, there was no lateral meniscus tear. Figure 5 shows the intact menisci. There were, however, grade one arthritic changes of the lateral femoral condyle and lateral tibial plateau. The lateral meniscus had a discoid shape. Everything was flushed out with saline. We closed with Monocryl sutures (Ethicon, Inc., Raritan, New Jersey, United States). Intra-operational arthroscopic images were obtained. Local anesthetic was placed around the incisions. Sterile dressings were placed including a compression dressing. The patient was awakened and was taken to the post-anesthesia care unit in stable condition.

Post-operatively, weight-bearing was advised as tolerated along with baby aspirin for two weeks. The patient was seen sixteen days after the surgery in the clinic with pain improvement and independent ambulation. Histopathology came back positive for synovial osteochondromatosis. The patient was scheduled for a subsequent follow-up in six weeks.

## Discussion

Patients with synovial osteochondromatosis frequently experience pain, edema, a reduction in ROM, and occasionally palpable loose bodies [10]. The condition commonly affects more males than females in their third to fifth decades of life and is usually monoarthritic with a predilection towards larger joints, with the knee and hip being most commonly affected, but can occur in any synovial joint. In our case, the patient was a 62-year-old female, showing that although there is a preference towards middle-aged males, this pathology has a wide spectrum regarding who it can affect. In some cases, the disease can extend beyond the joint space [10]. The disease process begins when cartilaginous bodies breach the joint capsule and continue to proliferate, leading to extra-articular involvement. Additionally, synovial-lined structures outside the joint, such as tendon sheaths and bursae, can also be affected in some instances. In our case, we saw this with the interpretation of the second MRI, with mucoid degeneration of the ACL.

Synovial osteochondromatosis is more commonly a primary condition but can be secondary to other joint pathologies or trauma from which the loose bodies originate from osteophytes or osteochondral lesions [11]. In secondary synovial osteochondromatosis, components of articular cartilage, bone, and fibrin may be seen along with the loose bodies and mostly affect older adults [11]. Synovial osteochondromatosis can also be sub-grouped into three stages: early, transitional, and late, as described by Milgram [11]. An active synovium without loose bodies can be seen in the early stage with the transitional stage showing an active synovium with loose bodies, and the late stage showing an inactive synovium with loose bodies present [11].

As mentioned earlier, the greatest risk with untreated synovial osteochondromatosis is a progression toward malignancy with chondrosarcoma, which is a rare complication. Chondrosarcoma usually arises from recurrent synovial osteochondromatosis rather than from untreated cases, yet malignancy should still be ruled out with histopathology [2]. The diagnosis of synovial osteochondromatosis can present challenges, and it is often a time-consuming process. On average, there is a reported delay of five years from the onset of symptoms to reaching a definitive diagnosis [2]. This is also consistent with our case, as it went to the extent of being missed on the first scan. Diagnosis is difficult due to its rarity, non-specific symptoms, variable presentation, limitations of diagnostic imaging, the need for histopathological examination, and lack of awareness. Healthcare professionals may have limited experience with the condition, leading to delays or missed diagnoses. The overlapping symptoms with other joint conditions can make it even more difficult to differentiate. Diagnostic imaging may not provide definitive evidence, and multiple studies over time may be required. This warrants an understanding of this disease, with a need for healthcare professionals to keep it on their differential when treating patients with joint pain and effusions. Increased awareness and collaboration among clinicians, radiologists, and pathologists are crucial for accurate diagnosis and management.

## Conclusions

Although synovial osteochondromatosis is generally a benign condition, transformation to chondrosarcoma is still a possibility. Imaging may assist in identifying the loose bodies floating in the joint space, but this becomes much more difficult if the loose bodies are radiotransparent (chondromatosis). Calcified nodules (osteochondromatosis) are relatively less difficult to view on X-ray. T2 weighted MRI imaging can help identify synovial hyperplasia which can help clue clinicians in with other physical exam findings. Although an arthroscopy with histopathology is the gold-standard diagnostic modality, it is important to keep in mind that one or even two different types of imaging modalities may not be sufficient for synovial osteochondromatosis to make the differential. As seen in our case, it took three years after the pain from the onset of chondromatosis for suspicion to arise on MRI. Sometimes patients may not be so fortunate by the time it is noticed and, although uncommon, the formation of chondrosarcoma may already be in progress. If left untreated, patients can also develop severe osteoarthritis. Clinicians must not hesitate to diversify the imaging modalities in which they order, as a diagnosis may become more apparent in one versus the other. Another lesson to take from our case is to stay vigilant in regard to who can present with synovial osteochondromatosis. Although our patient did fit the expected location of the disease (larger joint, the knee in this case), she did not fit the expected gender or age group in which these processes arise. It is also worth noting our patient's past medical history of hypertension and hypothyroidism; however, there is no literature that connects either one to the formation of synovial osteochondromatosis. 
